# Trends in Congenital Syphilis Cases by Maternal Country of Birth, Spain, 2016–2024

**DOI:** 10.3201/eid3207.260146

**Published:** 2026-07

**Authors:** Victoria Hernando, Carmen Montaño, Ana Fernandez, Laura Molina, Guillermo Perez, Luis Viloria, Raquel Morales, Henar Marcos, Evelin Lopez-Corbeto, Paula Silvestre, Santiago Vicente, Olaia Perez-Martinez, Laura Montero, M. Isabel Barranco-Boada, Jesus Castilla, Pello Latasa, Eva Martinez, Ninoska Lopez, Daniel Castrillejo, Ana Roldan, Asuncion Diaz, STIs Group of National Epidemiological Surveillance Network

**Affiliations:** National Centre of Epidemiology, Carlos III Health Institute, Madrid, Spain (V. Hernando, A. Diaz); CIBER in Infectious Diseases (CIBERINFEC), Instituto de Salud Carlos III, Madrid (V. Hernando, A. Diaz); Dirección General de Salud Pública del Gobierno de Aragón, Servicio de Vigilancia en Salud Pública, Zaragoza, Spain (C. Montaño); Dirección General de Salud Pública y Atención a la Salud Mental, Servicio Vigilancia Epidemiológica, Oviedo, Spain (A. Fernandez); Dirección General de Salud Pública, Servicio de Epidemiología, Palma de Mallorca, Spain (L. Molina); Dirección General de Salud Pública, Servicio Canario de Salud, Servicio de Vigilancia y Prevención, Las Palmas de Gran Canarias, Spain (G. Perez); Dirección General de Salud Pública Servicio de Vigilancia Epidemiologica, Santander, Spain (L. Viloria); Dirección General Salud Pública, Toledo, Spain (R. Morales); Dirección General Salud Pública, Valladolid, Spain (H. Marcos); Centro de Estudios Epidemiológicos sobre las ITS y Sida de Cataluña, Badalona, Spain (E. Lopez-Corbeto); Dirección General de Salud Pública, Servicio de Vigilancia y Control Epidemiológico, Valencia, Spain (P. Silvestre); Servicio Extremeño de Salud, Dirección General de Salud Pública, Mérida, Spain (S. Vicente); Dirección Xeral de Saúde Pública, Servizo de Vixilancia Epidemiolóxica, Santiago de Compostela, Spain (O. Perez-Martinez); Dirección General Salud Publica, Subdirección General de Vigilancia en Salud Pública Programas de Vigilancia de Infecciones de Transmisión Sexual, Madrid (L. Montero); Consejeria de Salud, Murcia, Spain (M.I. Barranco-Boada); Instituto de Salud Pública de Navarra-IdiSNA, CIBERESP, Pamplona, Spain (J. Castilla); Dirección General de Salud Pública, Servicio de Epidemiologia y Vacunación, San Sebastian, Spain (P. Latasa); Dirección General de Salud Pública, Consumo y Cuidados, Logroño, Spain (E. Martinez); Consejería de Sanidad, Servicio de Epidemiologia, Ceuta, Spain (N. Lopez); Dirección General de Salud Pública, Servicio de Vigilancia Epidemiológica, Melilla, Spain (D. Castrillejo); Consejería de Sanidad, Presidencia y Emergencias, Servicio de Vigilancia y Salud Laboral, Sevilla, Spain (A. Roldan)

**Keywords:** congenital syphilis, bacteria, sexually transmitted infections, surveillance, antenatal care, prevention, migrant, Spain

## Abstract

The number of congenital syphilis cases in Spain remains low; 40 cases were confirmed during 2016–2024. However, a slight increase has been observed, particularly in children born to migrant mothers. Young maternal age, migrant status, and social disadvantages are warning signs that underscore the need to strengthen prenatal screening.

Congenital syphilis is preventable through antenatal screening and treatment of pregnant women (*1*); however, a resurgence of congenital syphilis has been observed (*2*). During 2018–2022, rates surged by 599% in Canada (*3*) and 81.8% in the United States (*4*); slight increases were also noted in Europe (*5*). That increase stems from multiple factors, including limited healthcare access, substance use, and migration (*6*), which can introduce legal, cultural, and language barriers that heighten vulnerability (*7*). We analyzed trends in congenital syphilis in Spain during 2016–2024 by maternal country of birth.

## The Study

We analyzed confirmed cases of congenital syphilis in children <2 years of age reported to the National Epidemiologic Surveillance Network. In Spain, congenital syphilis has been a notifiable disease since 1997, although changes were made to the maternal variables collected in 2016. We categorized cases according to maternal country of birth: women born in Spain and women born elsewhere. We calculated annual incidence rates per 100,000 live births for each group using the annual number of live births in Spain as the denominator. We used the χ^2^ test to compare proportions and the Kruskal-Wallis test to compare medians.

Forty confirmed cases were reported during the study period. One case was excluded because of missing information on maternal country of birth. Of the 39 remaining cases, 12 (30.8%) cases involved children born to women born in Spain, whereas 27 (69.2%) cases involved infants of women born elsewhere (Figure). All children were born in Spain; no cases were classified as imported. From 2020 onward, incidence rates increased among women born outside Spain.

**Figure:**
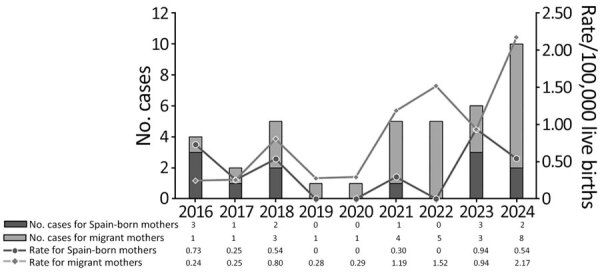
Number of congenital syphilis cases and rates per 100,000 live births by mother’s place of birth, Spain, 2016–2024.

Of the infants born to women born in Spain, 75.0% (n = 9) were boys, compared with 44.4% (n = 12) for women born elsewhere (p = 0.096). Median age at diagnosis was 2 days in both groups; interquartile range (IQR) was 1–28 days for children of women born in Spain and 1–7 days for children of women born elsewhere (p = 0.817). Symptoms were present in 50.0% (n = 6) of infants of women born in Spain and 55.5% (n = 15) of infants of women born elsewhere (p = 0.950) (Table 1). No differences were observed in clinical manifestations.

**Table 1:** Clinical manifestations of congenital syphilis cases by mother’s place of birth, Spain, 2016–2024

Manifestation	No. (%) cases		p value*
	Child born to woman born in Spain	Child born to woman born outside Spain	
Symptomatology			
Asymptomatic	2 (16.7)	4 (14.8)	0.950
Symptomatic	6 (50.0)	15 (55.6)	
Unknown	4 (33.3)	8 (29.6)	
Clinical manifestations, symptomatic cases only†			
Hepatosplenomegaly	4 (66.7)	8 (53.3)	0.577
Mucocutaneous lesions	3 (50.0)	4 (26.7)	0.306
Anemia	3 (50.0)	3 (50.0)	0.169
Central nervous system involvement	1 (16.7)	5 (33.3)	0.445
Jaundice	3 (50.0)	2 (13.3)	0.075
Nephrotic syndrome	1 (16.7)	1 (6.7)	0.481
Others	3 (50.0)	9 (60.0)	0.676

*χ^2^ was used to calculate p values. †Clinical manifestations are nonexclusive categories.

Hospitalization percentages were similar in both groups: 83.3% (n = 10) among infants of women born in Spain and 81.5% (n = 22) among infants of women born elsewhere (p = 0.792). Complications were also comparable, occurring in 25.0% (n = 3) of children born to women in Spain and 22.2% (n = 6) of children born to women born elsewhere (p = 0.241). Two deaths were reported; both were children born to women born in Spain.

A total of 38 women were included in this analysis; 11 (28.9%) were born in Spain and 27 (71.0%) outside Spain. One woman born in Spain gave birth to twins; congenital syphilis was diagnosed in both. Among women born outside Spain, most originated from countries in Latin America (n = 22), including Paraguay (n = 8) and Colombia (n = 4). Three were from Eastern Europe and 1 from Western Europe; in 1 case, country of origin was not specified.

Women born in Spain were younger at the time of delivery than women born elsewhere (Table 2). Syphilis screening had not been performed during pregnancy in 9.1% (n = 1) of women born in Spain and 11.1% (n = 3) of women born elsewhere. Adequate treatment was recorded for 36.4% (n = 4) of women born in Spain and 22.2% (n = 6) of women born elsewhere. Of the 39 women included in the analysis, 11 were living in socially or economically disadvantaged circumstances. In 1 case, the mother’s syphilis was diagnosed after the diagnosis of congenital syphilis in the child; that woman was born in Spain.

**Table 2:** Maternal characteristics in study of trends in congenital syphilis cases by maternal country of birth, Spain, 2016–2024*

Characteristic	Value		p value†
	Mother born in Spain†	Mother born outside Spain	
Total no. women	11 (28.9)	27 (71.1)	
Median age at delivery (IQR), y	21.5 (19–33)	25 (22–29)	0.421
Syphilis screening during pregnancy			0.877
First trimester	3 (27.3)	8 (29.6)	
Third trimester	0	2 (7.4)	
First and third trimester	2 (18.2)	2 (7.4)	
Not documented	2 (18.2)	4 (14.8)	
Not performed	1 (9.1)	3 (11.1)	
Unknown	3 (27.3)	8 (29.6)	
Syphilis treatment			0.284
Without treatment	4 (36.4)	11 (40.7)	
Appropriate treatment	4 (36.4)	6 (22.2)	
Inappropriate treatment	0	4 (14.8)	
During labor or delivery	3 (27.3)	2 (7.4)	
Not documented	0	3 (11.1)	
Unknown	0	1 (3.7)	
Maternal risk for syphilis			0.318
Yes	6 (54.6)	8 (29.6)	
None	1 (9.1)	6 (22.2)	
Unknown	4 (36.4)	13 (48.2)	
Risk factors, women with maternal risk for syphilis‡			
Disadvantaged situation	2 (33.3)	8 (100)	0.006
Engagement in sex work	1 (16.7)	1 (12.5)	0.825
Drug use	3 (50.0)	1 (12.5)	0.124
Unspecified factors	4 (66.7)	1 (12.5)	0.036
Maternal HIV coinfection			0.430
Yes	0	0	
No	9 (81.8)	23 (85.2)	
Not testing	0	2 (7.4)	
Unknown	2 (18.2)	2 (7.4)	

*Values are no. (%) except as indicated. †χ^2^ was used to calculate p values. ‡Risk factors are nonexclusive categories.

## Conclusions

Although the absolute number of congenital syphilis cases remains low in Spain, an increase has been observed in recent years, particularly among women born outside Spain. The observed increase in cases might be considered a consequence of the rising number of syphilis cases among young women. Previous studies in Spain have shown that the greatest increases in syphilis occurred among women 20–34 years of age (*8*) and women from Latin America (*9*). However, data are unavailable on the percentage of women with syphilis who were pregnant at the time of diagnosis or whose condition was diagnosed during delivery.

Of the confirmed congenital syphilis cases in this study, two thirds were in infants of women born outside Spain, whereas pregnancies among women born elsewhere accounted for only 25.6% of all pregnancies in 2024 (*10*). That difference might indicate unmet needs in antenatal care for migrant women, which pose risks to both child and maternal health, even though access to pregnancy, childbirth, and postpartum care is guaranteed to all women in Spain regardless of administrative status (*11*). Therefore, ensuring that migrants receive comprehensive information regarding their own health and prenatal care during pregnancy, such as screening tests, is essential to guaranteeing adequate follow-up care.

Most women in this study originated from countries in Latin America, which also represents the largest region of origin among migrants in Spain (*10*). Migration during pregnancy is not rare and poses a challenge for timely diagnosis and treatment of syphilis in pregnant women.

Other factors, such as younger maternal age, might also contribute to increased vulnerability. National data show a rising trend in maternal age in Spain. Among women born in Spain, the mean age at delivery increased from 32.5 years in 2016 to 33.1 years in 2023, whereas among women born elsewhere it rose from 29.5 years to 30.5 years over the same period (*10*). In contrast, the mothers in this analysis were younger; median age was 21.5 years for women born in Spain and 25 years for women born elsewhere.

Adverse birth outcomes related to congenital syphilis are closely linked to inadequate access to timely screening and treatment during pregnancy (*12*). Screening has also been shown to reduce syphilis-related deaths by up to 50% (*13*). In this analysis, the percentage of women whose condition was adequately diagnosed and treated was very low in both groups, underscoring the need for improved implementation of existing recommendations. Serologic screening for syphilis during pregnancy is recommended at the first prenatal visit, and additional testing should be performed during the third trimester and at delivery for women at high risk of acquiring syphilis (e.g., because of sex work, drug use, disadvantaged social conditions, or other sexually transmitted infections during pregnancy) or if no test was performed during the first trimester (*14*,*15*). Although information on predisposing factors was available for a limited number of women, more than half (14/26) were in disadvantaged situations, highlighting the role of broader social determinants of health.

Epidemiologic surveillance data provide a comprehensive overview of trends in the general population but have limitations. Under case definitions in Europe, only children <2 years of age are included, and fetal deaths or stillbirths caused by congenital syphilis are excluded, potentially leading to underdiagnosis. In addition, maternal information in surveillance records is often incomplete. Enhancing those data could help elucidate existing gaps in prenatal care for pregnant women and identify groups that are more vulnerable to syphilis.

Our findings highlight the importance of ensuring timely, accessible, and respectful prenatal care and syphilis screening for all pregnant women, particularly when social or structural vulnerabilities are present. Strengthening epidemiologic surveillance and reinforcing person-centered approaches in maternal care are essential steps toward preventing congenital syphilis and promoting maternal and child health.
